# Minimally Invasive Endovascular Management of Uterine Arteriovenous Malformations: A Single Center Experience and Case Series

**DOI:** 10.7759/cureus.62156

**Published:** 2024-06-11

**Authors:** Islam Kourampi, Nityanand Jain, Piyush Chaudhary, Ravul Jindal

**Affiliations:** 1 Department of Medicine, National and Kapodistrian University of Athens School of Medicine, Athens, GRC; 2 Department of Vascular Surgery, Fortis Hospital, Mohali, IND

**Keywords:** glue, pregnancy, uterine, vaginal bleeding, arteriovenous malformation, uae, uterine artery embolization

## Abstract

Uterine arteriovenous malformations (UAVMs) are rare and abnormal entanglements of uterine arteries and veins that are potentially fatal, requiring blood transfusions in about a third of cases. Although the optimal management of the condition is not well established in the literature, surgical hysterectomy is believed to be the only definitive treatment for arteriovenous malformations. We present three cases of UAVMs treated by a minimally invasive endovascular approach. Chief complaints were heavy menstrual bleeding and sudden onset heavy bleeding. The diagnosis was confirmed by computed tomography imaging and angiography of the pelvic vessels. Uterine artery embolization (UAE) was performed in all patients. The follow-up period was uneventful. In our experience, the UAE provides satisfactory results in terms of success rates, complications, and short hospital stays.

## Introduction

Uterine arteriovenous malformations (UAVMs) are potentially life-threatening peripheral tangles of blood vessels that disturb the blood flow and oxygen transport to the local tissue microenvironment [1]. They may be congenital (due to the failure of embryological development in the primitive capillary plexus) or acquired (due to infections, choriocarcinoma, or uterine interventions such as curettage or surgery) [1,2]. Since its first description in the early 1900s, less than 150 cases have been reported in the literature, raising suspicion about its true incidence [3]. UAVMs can be fatal due to severe bleeding and the need for a blood transfusion in up to 30% of the patients [4]. Vaginal bleeding (menorrhagia, metrorrhagia, and menometrorrhagia) and localized abdominal pain have been reported as presenting symptoms, which can often be seen as severe anemia in blood analysis [5,6]. It is recommended to maintain a heightened index of suspicion for UAVM in the case of young multiparous women and in those with a history of fetal loss or endometrial intervention who present with the aforementioned symptoms, especially since a timely diagnosis is essential for effective patient management [7,8]. A safe and non-invasive initial test is the Doppler ultrasound, which shows turbulent blood flow and multiple entangled feeding vessels. Pelvic computed tomography (CT), magnetic resonance imaging (MRI), and angiography can highlight vascular pathology and enhance the diagnostic approach [1]. Management is guided primarily by hemodynamic stability and the desire to preserve fertility. An endovascular approach should be considered in patients with hemodynamic instability, anemia, or recurrent episodes of metrorrhagia. On the other hand, hemodynamically stable patients could be managed conservatively [8,9]. In this context, we present a case series of three patients with UAVMs who were successfully treated with uterine artery embolization (UAE). The article was previously presented as a meeting abstract at the 12th Munich Vascular Conference 2023, Germany, on December 7-8, 2023.

## Case presentation

Patient 1

**Figure 1:**
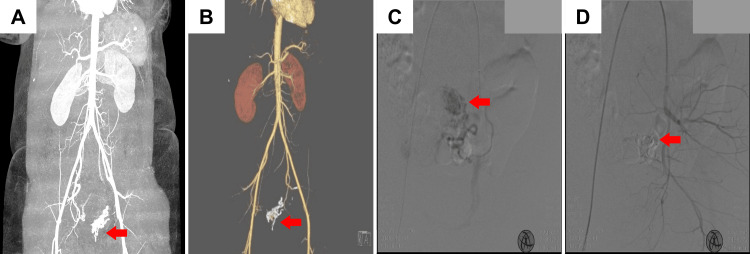
Computed tomography (CT) and angiogram of patient 1 (A, B) CT pelvis showing the UAVM (red arrow) involving the left lateral uterine wall and the endometrium. (C) Super-selective cannulation of the distal uterine artery was performed using the microcatheter. The angiogram shows the arteriovenous communications (red arrow) arising from the left uterine artery. (D) Post-uterine artery embolization shows complete occlusion (red arrow) of the UAVM and glue cast. UAVM: uterine arteriovenous malformation

A 31-year-old female patient presented to our department with complaints of major vaginal bleeding over the past five days despite urgent hemostatic packing in the emergency department. The patient had a history of hypothyroidism (managed with medication), allergic rhinitis, and a lower-segment C-section performed two years ago. An episode of mild bleeding for the last three months post-dilation and curettage (D&C) performed for abortion was reported by the patient. The physical exam was unremarkable except for active vaginal bleeding. The vitals were normal at admission (Table 1). A CT scan of the pelvis revealed UAVM involving the left lateral uterine wall and the endometrium (Figure 1).

**Table 1:** Baseline patient vitals and investigations conducted at the time of admission ↓indicates blood value lower than normal range value while N indicates blood value within normal range. UAVM: uterine arteriovenous malformation

Parameters	Case 1	Case 2	Case 3
Patient age (years)	31	33	32
No. of hospitalized days	2	1	1
Diagnosis	Left UAVM	Bilateral UAVM	UAVM with bilateral supply
Patient vitals at admission			
Blood pressure (mmHg)	130/80	110/70	120/80
Temperature (^0^F)	98.6	98.2	98.0
Pulse (x/min)	80	78	78
Respiratory rate (x/min)	18	20	18
SpO_2_ on room air (%)	98	98	100
Physical examination at admission			
Abdominal examination	Normal	Normal	Painful palpations
Thoracic examination	Normal	Normal	Normal
Peripheral examination	Normal	Normal	Normal
Vaginal examination	Active localized bleeding	Active localized bleeding	No active bleeding
Laboratory investigations at admission			
Hemoglobin (g/dL)	9.80 (↓)	7.10 (↓)	12.10 (N)
Red blood cell count (million/µl)	3.31 (↓)	3.52 (↓)	4.72 (N)
Leukocyte count (thousand/µl)	8.81 (N)	8.50 (N)	8.72 (N)
International normalized ratio (INR)	0.95 (N)	0.96 (N)	0.95 (N)

Based on the radiological findings, an emergency UAE was done. Under general anesthesia, retrograde arterial access was achieved from the right femoral artery for arterial embolization using a 6F sheath. The patient was given 3000 IU of heparin during the procedure. The angiogram confirmed the presence of UAVM arising from the left uterine artery (Figure 1). Selective embolization of the vessel was achieved using endocryl glue and lipiodol. A post-embolization angiogram revealed remission of the AVM and occlusion of the left uterine artery. Postoperatively, the patient received intravenous (IV) fluids, antibiotics, and supportive treatment. The patient was discharged with amoxicillin/clavulanic acid 625 mg and acetaminophen 500 mg three times daily for three days. One year follow-up was unremarkable, and the patient had a successful recovery with stabilization of anemia.

Patient 2

**Figure 2:**
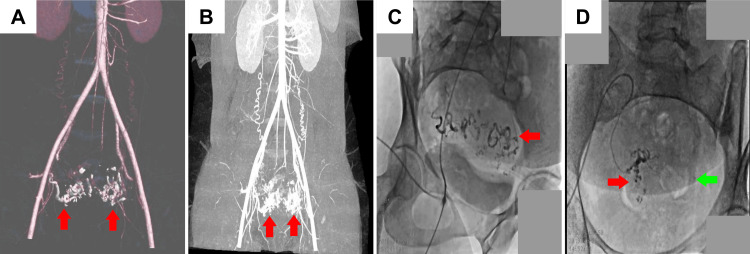
CT angiography for patient 2 (A, B) CT pelvis showing bilateral UAVM (red arrows) involving the left and right uterine wall. (C) Glue cast confirming the presence of UAVM arising from the left uterine artery. (D) Post-uterine artery embolization glue cast showing embolization of the left UAVM (green arrow) and injection of endocryl glue with lipiodol through microcatheter on the right side (red arrow). UAVM: uterine arteriovenous malformation

A 33-year-old female patient was admitted to our department for vaginal bleeding which was very frequent during the past three weeks. The patient reported using two sanitary pads daily for two weeks which increased to five pads daily in the week preceding her presentation to our department. The patient's medical history indicated a lower-segment C-section performed three years ago. A pelvic MRI done at another center showed a lobulated hyperintense lesion of the right fundal region of the uterus with myometrial involvement and endometrial extension. CT angiography depicted early enhancement and washout with increased vascularity in the uterine wall and parametrium (Figure 2). Suspicion of UAVM was established. Due to the lack of vein enhancement, in the differential diagnosis, other hyper-vascular uterine lesions like gestational trophoblastic tumors and aggressive neoplasms were also considered. The patient was referred for an oncological consultation with the on-call oncologist. The blood test for human chorionic gonadotropin (HCG) was negative and after the oncologist's evaluation, neoplasms were ruled out. No biopsy was taken considering the risk of bleeding due to the hypervascular nature of the lesion. Subsequently, the patient was planned to be managed endovascularly. Under general anesthesia, vascular access was accomplished via the right common femoral artery, and a 5F sheath insertion was done. 5000 IU heparin was administered during the procedure.

**Figure 3:**
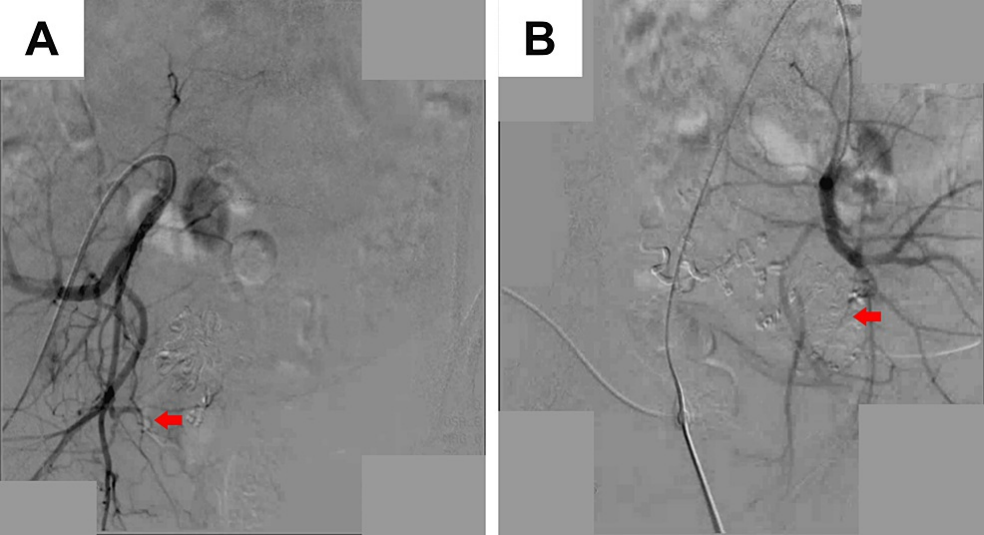
Angiogram for patient 2 (A) Check angiogram showing good closure of right UAVM arising from the right internal iliac artery (red arrow). (B) Check angiogram showing good closure of the left UAVM arising from the left internal iliac artery (red arrow). UAVM: uterine arteriovenous malformation

After obtaining a selective right internal iliac artery angiogram, a UAVM of the right uterine artery draining to a large vein was demonstrated. The right uterine artery was selectively catheterized. A right uterine artery angiogram was then performed, and glue embolization of the vessel was done until blockage of the vessel was attained (Figure 3). The left iliac artery was catheterized, and an angiogram was performed, where an AVM filling from the left uterine artery was observed (Figure 3). Glue was injected until occlusion of the left uterine artery was achieved. After bilateral UAE, a pelvic angiogram was carried out to confirm post-embolization stasis of flow in both uterine arteries. Postoperatively the patient had an uneventful recovery and was discharged the next day. The patient continued to receive intervaginal packing for four days and reported using two sanitary pads daily. No bleeding episodes were reported by the patient from the fifth day onwards. The patient was recommended regular follow-up checkups for one year. However, the patient was lost to follow-up.

Patient 3

**Figure 4:**
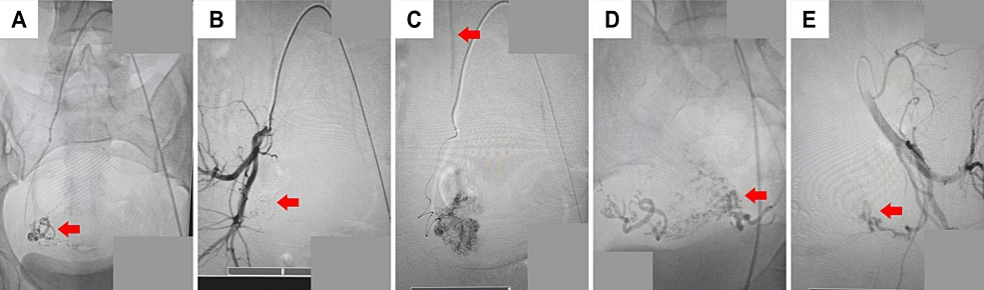
Angiogram for patient 3 (A, B) Angiogram showing the UAVM (red arrow) involving the right lateral uterine wall before and after embolization. (C) Angiogram demonstrating the draining vein (red arrow) into the UAVM. (D, E) Angiogram confirming the presence of UAVM (red arrow) arising from the left uterine artery before and after embolization.

A 32-year-old female presented to the emergency department with severe right-sided abdominal pain and episodes of on-and-off bleeding (spotting). The patient reported that spotting began post-D&C for abortion carried out a month earlier. There was no significant family history of gynecologic or genetic disease. The angiogram showed a UAVM with bilateral supply (Figure 4). It was not clear at the time whether the UAVM was a pre-existing condition before D&C or an iatrogenic complication. At our center, we have seen post-traumatic arteriovenous communications develop within a month in other body parts such as legs and arms, especially post-catheterization and in patients with gunshot bullet injuries. An endovascular arterial embolization was undertaken for this patient. Under general anesthesia, a left groin puncture was done, and left common femoral artery access was obtained, where a 6F sheath was placed. Then, 5000 IU heparin was administered to prevent clotting and blockage of the catheter. Perioperative selective angiography of both internal iliac arteries was conducted (Figure 4). It revealed a hyper-vascular endometrial AVM fed by dilated bilateral uterine arteries with an early draining vein. The left uterine artery was selected and subsequently embolized using glue. The same procedure was performed on the right uterine artery. Postoperatively the patient was shifted to the ward and was given supportive treatment. The patient was discharged in stable condition and followed up regularly as an outpatient for the next two years. No adverse events were noted in this follow-up period.

## Discussion

UAVMs are a rare condition that affects women of both reproductive and non-reproductive ages. Its incidence reportedly ranges from 0.1% to 2% but is expected to increase due to the rising rate of uterine manipulations, such as D&C, abortion, and C-sections [4,10,11]. UAVMs are typically identiﬁed in symptomatic, multiparous women of childbearing age and/or in nulliparous women after instrumentation. Hence, taking proper history is very important as it may lead us to etiology. The patients may present with either asymptomatic mass or large pulsatile masses, recurrent pregnancy loss, congestive heart failure caused by significant arteriovenous shunting, hypotension, anemia, menorrhagia, or life-threatening bleeding [12,13]. UAVMs may be misdiagnosed as incomplete abortion or gestational trophoblastic disease (GTD) in women who experience recurrent vaginal bleeding after pregnancy termination or post-C-section. Occasionally, UAVMs co-occur with retained products of conception, which can be distinguished using beta-human chorionic gonadotropin (β-hCG) levels and imaging modalities [13]. It is crucial not to base suspicion of UAVM on the duration of complaints alone as exemplified by the case of a 40 years old female patient with an 18-year long history of recurrent vaginal bleeding [14]. Instead, suspicions must be guided by a combination of patient history, volume of blood loss, complicating features, and overall clinical condition. Other potential indications are non-specific pelvic discomfort, urinary symptoms like excessive urination and incontinence, dyspareunia, as well as pelvic pain or pressure, including neuropathic pain like sciatica [3,15].

Before modern imaging techniques were widely available, diagnosis of UAVM was done by laparotomy or after hysterectomy. However today, an initial screening can be performed using ultrasonography (US). Although the diagnostic accuracy of US relies on the technician’s proficiency and machine capabilities, it is an affordable, non-invasive, and non-ionizing diagnostic tool for excluding differentials like GTD, hyper-vascular lesions, endometrial hyperplasia, oncological lesions, fibroids, and pseudoaneurysms. Characteristics of UAVMs on grayscale sonography include a poorly defined, non-homogenous mass of hypoechoic cystic or tubular-like structures of varying sizes. Additionally, there is a focal or asymmetric thickening of the myometrium and endometrium. The vascular nature is confirmed by Doppler examination, which shows multidirectional, high-velocity flow and color mosaic patterns due to color aliasing and apparent flow reversal [1]. Uterine artery digital subtraction angiography (DSA) remains the gold standard for diagnosing and treatment planning of UAVMs. This is due to its unmatched ability to identify and localize vascular nidus along with the assessment of the size and flow of the arteriovenous shunt associated with the AVM. The hallmark of UAVMs is the rapid and early enhancement of multiple dilated veins arising from a nest of anomalous vessels following the administration of arterial contrast. For larger AVMs, CT angiography or MR angiography (MRA) may be an adjunct to diagnosis and treatment planning [1]. In our experience, dynamic contrast-enhanced MRA is more effective than CT in displaying the size and reach of the lesion. It also has the benefit of being able to reveal disruption of the junctional zone and endometrial involvement. Other advantages of MRA include improved characterization of pelvic organs and the absence of ionizing radiation, which may be especially important for women of reproductive age. After an accurate diagnosis of UAVM, the treatment approach will depend on the patient's age, hemodynamic stability, degree of bleeding, size, and site of UAVM, and desire for future pregnancy. The literature offers little consensus regarding the optimal management approach.

However, decisions between conservative or surgical alternatives are made based on clinician experience and conclusions drawn from published case reports. In a systematic review that identified all available literature on iatrogenic acquired UAVMs, UAE was the most frequent treatment option, utilized in 59% of cases, followed by hysterectomy at 29% [16]. Spontaneous resolution occurred in only 6% of patients. About 17% of patients who received UAE experienced recurrence. Recovery was reportedly fast and viable even in patients with anemia, high risk, or hemodynamic instability [16]. UAE has also been found to be associated with reduced hospitalization time and lower rates of complications [17]. In the UAE procedure, selective catheters are guided percutaneously to the proximal end of the feeders, allowing for the introduction of embolic agents. Microcatheters are used to cannulize smaller feeders close to the nidus for more effective embolization. At our department, we performed UAE procedures in all patients under general anesthesia due to patient apprehension and fear, and most importantly to reduce the feeling of intense stinging pain when the endocryl glue is injected. This provides a superior procedural experience for the patient. Despite good closure shown in angiograms post trans-arterial embolization, even a small communication between the artery and vein can lead to UAVM persistence or recurrence. In such instances, transvenous embolization, with or without percutaneous sclerotherapy, may be necessary. The risks of this procedure involve unintended delivery of embolic material. Regarding the embolic agent, a study of 14 patients who underwent UAE found that normal pregnancies were achieved regardless of the type of embolic agent used, indicating that the choice of embolic agent has no impact on future fertility [18]. In our reported cases, glue was used exclusively. However, polyvinyl alcohol, microfibrillar collagen, steel coil springs, and absorbable gelatine sponges have also been successfully used for embolization, especially when the malformation is located at a distance and further catheter advancement is difficult or impossible [3,19]. These products are sometimes used in combination for better occlusion. Alcohol may also be utilized in the UAE; however, its role is more as a sclerosing agent rather than an embolizing material. The most frequent side effect of UAE is pelvic pain or cramping, which can be self-limiting or managed with analgesics. In some rare cases, infection may occur after the embolization procedure which can be managed with appropriate antibiotics. Other complications comprise perianal skin shedding, uterovaginal and rectovaginal fistulas, and neurological deficits in the lower extremities [20]. Transvaginal ultrasound (TVUS) and MRA are recommended imaging modalities to evaluate the uterus after embolization.

## Conclusions

UAE is emerging as a leading treatment option for patients experiencing life-threatening vaginal bleeding in UAVM. It is particularly recommended for young patients and those who desire to preserve their fertility. UAE provides symptomatic relief with fewer side effects and complications and can greatly decrease morbidity and mortality if UAVM is identified and diagnosed early. Regular follow-up and check imaging are recommended after UAE for at least one year post-procedure.
